# Associations Between Heart Rate Variability Measured With a Wrist-Worn Sensor and Older Adults’ Physical Function: Observational Study

**DOI:** 10.2196/13757

**Published:** 2019-10-23

**Authors:** Sarah Anne Graham, Dilip V Jeste, Ellen E Lee, Tsung-Chin Wu, Xin Tu, Ho-Cheol Kim, Colin A Depp

**Affiliations:** 1 Sam and Rose Stein Institute for Research on Aging University of California San Diego La Jolla, CA United States; 2 Department of Psychiatry University of California San Diego La Jolla, CA United States; 3 Department of Neurosciences University of California San Diego La Jolla, CA United States; 4 Department of Mathematics University of California San Diego La Jolla, CA United States; 5 Scalable Knowledge Intelligence, IBM Research – Almaden San Jose, CA United States; 6 VA San Diego Healthcare System San Diego, CA United States

**Keywords:** wearable technology, aging, electrocardiogram, geriatric assessment

## Abstract

**Background:**

Heart rate variability (HRV), or variation in beat-to-beat intervals of the heart, is a quantitative measure of autonomic regulation of the cardiovascular system. Low HRV derived from electrocardiogram (ECG) recordings is reported to be related to physical frailty in older adults. Recent advances in wearable technology offer opportunities to more easily integrate monitoring of HRV into regular clinical geriatric health assessments. However, signals obtained from ECG versus wearable photoplethysmography (PPG) devices are different, and a critical first step preceding their widespread use is to determine whether HRV metrics derived from PPG devices also relate to older adults’ physical function.

**Objective:**

This study aimed to investigate associations between HRV measured with a wrist-worn PPG device, the Empatica E4 sensor, and validated clinical measures of both objective and self-reported physical function in a cohort of older adults living independently within a continuing care senior housing community. Our primary hypothesis was that lower HRV would be associated with lower physical function. In addition, we expected that HRV would explain a significant proportion of variance in measures of physical health status.

**Methods:**

We evaluated 77 participants from an ongoing study of older adults aged between 65 and 95 years. The assessments encompassed a thorough examination of domains typically included in a geriatric health evaluation. We collected HRV data with the Empatica E4 device and examined bivariate correlations between HRV quantified with the triangular index (HRV TI) and 3 widely used and validated measures of physical functioning—the Short Physical Performance Battery (SPPB), Timed Up and Go (TUG), and Medical Outcomes Study Short Form 36 (SF-36) physical composite scores. We further investigated the additional predictive power of HRV TI on physical health status, as characterized by SF-36 physical composite scores and Cumulative Illness Rating Scale for Geriatrics (CIRS-G) scores, using generalized estimating equation regression analyses with backward elimination.

**Results:**

We observed significant associations of HRV TI with SPPB (n=52; Spearman ρ=0.41; *P*=.003), TUG (n=51; ρ=−0.40; *P*=.004), SF-36 physical composite scores (n=49; ρ=0.37; *P*=.009), and CIRS-G scores (n=52, ρ=−0.43; *P*=.001). In addition, the HRV TI explained a significant proportion of variance in SF-36 physical composite scores (R^2^=0.28 vs 0.11 without HRV) and CIRS-G scores (R^2^=0.33 vs 0.17 without HRV).

**Conclusions:**

The HRV TI measured with a relatively novel wrist-worn PPG device was related to both objective (SPPB and TUG) and self-reported (SF-36 physical composite) measures of physical function. In addition, the HRV TI explained additional variance in self-reported physical function and cumulative illness severity beyond traditionally measured aspects of physical health. Future steps include longitudinal tracking of changes in both HRV and physical function, which will add important insights regarding the predictive value of HRV as a biomarker of physical health in older adults.

## Introduction

### Background

Heart rate variability (HRV), or variation in beat-to-beat intervals of the heart, is a quantitative measure of autonomic regulation of the cardiovascular system that reflects the ability of the system to react to stressors [1-3]. Low HRV indicates improper coordination between the sympathetic and parasympathetic nervous systems and is a well-established predictor of future cardiovascular disease [4-8]. HRV is also linked to other aspects of health that are directly impacted by autonomic function such as self-regulatory capacity and psychological and physiological stress [5,9,10]. Most HRV metrics are shown to decline normally with age, primarily during younger decades, and some of these metrics increase again after the seventh decade [4,11-15]. In studies of older adults, low HRV measured by electrocardiogram (ECG) recordings has shown preliminary relationships with physical frailty [16-19]. Thus, measurement of HRV may add important information to an assessment of older adults’ physical functioning and help to identify individuals at higher risk for physical decline.

### Heart Rate Variability Measurement

HRV is traditionally measured in clinical or laboratory settings using standard ECG equipment or in the field with a 24-hour Holter monitor [20]. Although accurate, these measures are time consuming, require expert setup, and are obtrusive, making them less appropriate for health assessments and unlikely to be routine. Recent advances in technology enable measurement of HRV in more ecologically valid settings with unobtrusive wearable devices [21,22]. For example, wrist-worn devices capture HRV via photoplethysmography (PPG) sensors that detect blood volume changes in the microvasculature with each heartbeat [22-24]. These blood volume changes allow the determination of a PPG-derived peak-to-peak (P-P) interval, which is a valid proxy measure of the R-R interval derived from ECG recordings [25-27]. P-P or R-R intervals are also known as interbeat intervals and reflect the durations between successive heartbeats. HRV can be calculated from interbeat intervals with many different metrics (eg, time domain: standard deviation of normal-to-normal index; geometric: triangular index [TI]; and frequency domain: low frequency to high frequency power ratio) [3,20,28]. Selecting the appropriate HRV metric requires careful consideration of the length of recording and quality of the data. Wrist-worn PPG devices for HRV monitoring are increasing in popularity because of their ease of use; however, there is very little literature regarding the clinical use of these devices [29]. A critical step toward understanding the utility of these devices is to determine if and how HRV metrics derived from wrist-worn PPG devices relate to the physical function of older adults. To our knowledge, this is the first study using a wrist-worn PPG device to derive HRV in an older adult population.

### Study Purpose

If HRV metrics derived from a wrist-worn wearable are related to clinical measures of physical function and further explain variability in physical health status, a wearable tool could be a useful addition to regular clinical geriatric health assessments for older adults. The primary aim of this study was to investigate associations between HRV measured with a wrist-worn PPG device, the Empatica E4 sensor (Empatica Inc) [30,31], and widely used and validated clinical measures of physical function, including Short Physical Performance Battery (SPPB) scores, Timed Up and Go (TUG) scores [32-34], and self-reported physical function (SF-36 physical composite scores) [35], in a cohort of older adults living independently in a continuing care senior housing community (CCSHC). We selected the SPPB, TUG, and SF-36 as objective and subjective measures of physical capacity and function, respectively, because they are a few of the most widely used measures of physical function in clinical geriatric research and health assessments [36-39]. Our primary hypothesis was that lower HRV would be associated with lower physical function. We further investigated whether HRV could explain additional variability in physical health status, as measured by the SF-36 physical composite scores and the presence and severity of physical comorbidities via the Cumulative Illness Severity Scale for Geriatrics (CIRS-G) [40], beyond traditionally documented aspects of health such as age, gender, race, blood pressure, medication and alcohol use, smoking status, and anthropometric measurements.

## Methods

### Participants

The University of California San Diego (UCSD) Human Research Protections Program (HRPP) approved the study protocol. Research staff recruited participants from a CCSHC in San Diego County via short presentations using an HRPP-approved script and flyers. We recruited 77 participants living in the independent living sector of the CCSHC, and all of them provided written informed consent before study participation. The inclusion and exclusion criteria for enrollment were (1) English-speaking individuals older than 65 years, (2) ability to complete study assessments, and (3) no known diagnosis of dementia or other disabling illness.

### Procedures

We evaluated participants during the baseline assessment in an ongoing study of older adults between 65 and 95 years of age [41]. This assessment encompassed a thorough examination of domains typically included in a geriatric health evaluation: sociodemographic and medical health information, physical function measurements, cognitive measurements, and additional assessments of everyday function. The mean duration of the assessments was 2:34:31 (HH:MM:SS; range 1:09:30-4:38:10). The range of activities performed during this assessment reflected the types of tasks that an individual may encounter in their daily lives, and thus, the measure of HRV obtained from this time period is more similar to HRV metrics obtained over longer durations (eg, 24 hours), reflecting the cardiovascular system’s response to a range of environmental stimuli and workloads, as opposed to short-term measurements (eg, <5 min) that reflect immediate responses to a particular stimulus [3].

For this investigation, we characterized participants with sociodemographic and clinical information including age, sex, race, education, body mass index (BMI), waist-to-hip ratio, blood pressure, medication use (antihypertensives and antidepressants), presence and severity of physical comorbidities reported with the CIRS-G [40], and capacity for everyday functioning with the UCSD Performance-Based Skills Assessment Brief [42].

### Physical Function Measurement

We assessed physical functioning with both objective, capacity-based measures and subjective, self-reported measures. Capacity-based measures included the SPPB and TUG test, both of which have been shown to be valid, objective measures of lower extremity function and mobility [32-34]. The SPPB has 3 subcategories: the ability to stand for 10 seconds with feet in 3 different positions (side by side, semitandem, and tandem), 2 timed trials of a 3- or 4-m walk (faster of the two), and the time it takes to rise from a chair 5 times. Scores range from 0 to 12, with higher scores indicating better lower extremity function. The TUG test is scored by the amount of time it takes to rise from a chair, walk 3 m at a comfortable pace, turn, return to the chair, and sit down. A shorter time to complete the test indicates better mobility. For self-reported physical functioning, we used the physical composite score from the Medical Outcomes Study Short Form 36 (SF-36), which is a globally used questionnaire for assessing 8 dimensions of health-related quality of life [35]. The physical composite score is an aggregate of the 8 scale scores reflecting self-reported physical health [43].

### Heart Rate Variability Measurement

We collected raw PPG signals and interbeat intervals for the calculation of HRV with a wrist-worn device called the Empatica E4 [30,31]. Research staff placed the Empatica E4 wristband on a participant’s nondominant wrist at the onset of the assessment. During the first few minutes, the staff member checked the quality of the PPG signal in real time through the E4 application to ensure proper wrist placement as recommended by Empatica. Wrist-worn PPG sensors tend to be more accurate at rest than during exercise because of contamination from movement artifacts and often require accelerometry technology to measure consistent or repetitive movements to minimize influence of these artifacts [21,44-47]. The PPG sensor in the Empatica E4 is designed to be robust against movement artifacts in that it can attenuate noise even when movements are not repetitive in nature, using an artifact removal technique based on a combination of multiple infrared light wavelengths [30].

The Empatica E4 provided continuous heart rate and interbeat intervals that were not interpolated. It removed interbeat intervals that corresponded to regions where the PPG signal was not clear. For postprocessing, we downloaded the interbeat intervals provided in a comma-separated value file format from the E4 connect application. We viewed each interbeat interval time series in MATLAB R2016a (MathWorks, Inc) and calculated the percentage of gaps in the data (ie, nonconsecutive interbeat intervals) for each participant. We removed 19 participants from data analyses because of poor-quality interbeat interval recordings with greater than 20% gaps between interbeat intervals that resulted in less than 20 min of recorded high-quality interbeat interval data. We then processed the remaining data with Kubios HRV standard software version 3.1.0 using a low threshold artifact correction to adjust for any remaining ectopic beats and the smoothing priors detrending method (default λ=500) [48,49].

Selecting an HRV metric requires careful consideration of the strengths and limitations of wrist-worn PPG devices. The Empatica E4 uses algorithms to remove errant interbeat intervals and provides the remaining *clean* data that contain regions where interbeat intervals are not necessarily consecutive [30,31,50]. To overcome this limitation and provide a robust and valid HRV metric, we selected a metric that is less sensitive to gaps between interbeat intervals called the HRV TI [44,51,52]. The HRV TI is a geometric index calculated as the integral of the density distribution of interbeat intervals divided by the maximum of the density distribution [28] with larger numbers indicating more favorable HRV. The HRV TI requires a longer recording period (approximately 20 min), is robust against missing interbeat intervals, and has good intraindividual reproducibility [28]. For these reasons, we selected the HRV TI and analyzed the longest recording period available for each participant (ie, full-length assessment) for greater measurement stability.

### Statistical Analyses

Given the exploratory nature of this study, we did not perform a formal a priori power analysis. We performed correlational statistical analyses in SPSS version 25 and generalized estimating equation (GEE) regression with backward elimination in R version 3.4.1. We used Spearman correlation coefficients to assess bivariate correlations between the HRV TI measured across the full assessment period and SPPB, TUG, and SF-36 physical composite scores. We removed an additional 5 participants from correlational analyses for the suspected presence of arrythmia based on an HRV TI greater than 20.42 [53]. We were also missing SPPB scores for 1 participant, TUG scores for 2 participants, and SF-36 physical composite scores for 4 participants. We included the remaining participants in analyses (n=52, SPPB; n=51, TUG; and n=49, SF-36 physical composite). We conducted Mann-Whitney U tests to determine if there were differences between the participants removed from analyses and those retained. For our primary analyses, we first examined correlations between the HRV TI and variables that have been previously shown to be related to HRV including age, BMI, blood pressure, and mean heart rate [4,54]. We also looked for group differences in the HRV TI between men and women and between participants on versus off antihypertensive and antidepressant medications [16]. We used these results to determine whether to adjust for any of these variables using partial correlations (ie, control for one or more of these variables using nonparametric partial correlation syntax in SPSS if any covariates could potentially modify our primary bivariate relationships of interest). For the regression analyses, we included n=42 participants with complete data on all potential covariates (age, gender, race, blood pressure, BMI, waist-to-hip ratio, SPPB and TUG scores, antihypertensive and antidepressant use, alcohol use, smoking status, SF-36 physical composite or CIRS-G scores depending on the model, and HRV). We checked variance inflation factors (VIFs) for each covariate and considered a covariate with VIF greater than 3 as having high multicollinearity [55]. We rebuilt linear models and recalculated VIFs for each covariate after excluding covariates with a VIF greater than 3 and repeated this exclusion process until all covariates had a VIF less than 3. To build the regression model, we started with all variables of VIF less than or equal to 3 in the GEE model [56]. We then iteratively removed the variable with the largest *P* value, rebuilt a new GEE model based on the remaining variables, and recalculated *P* values for each variable. We repeated this process until all remaining variables had a *P* value at a threshold of less than or equal to .2. The backward elimination procedure ensured minimum bias in the final model [57]. We derived *R^2^* values using the linear model. We established statistical threshold for each family of statistical tests: correlational significance to a Bonferroni adjusted *P*≤.017 (0.05/3 primary outcomes of interest) and *P*≤.025 for the regressions (0.05/2 regression analyses).

## Results

**Figure 1:**
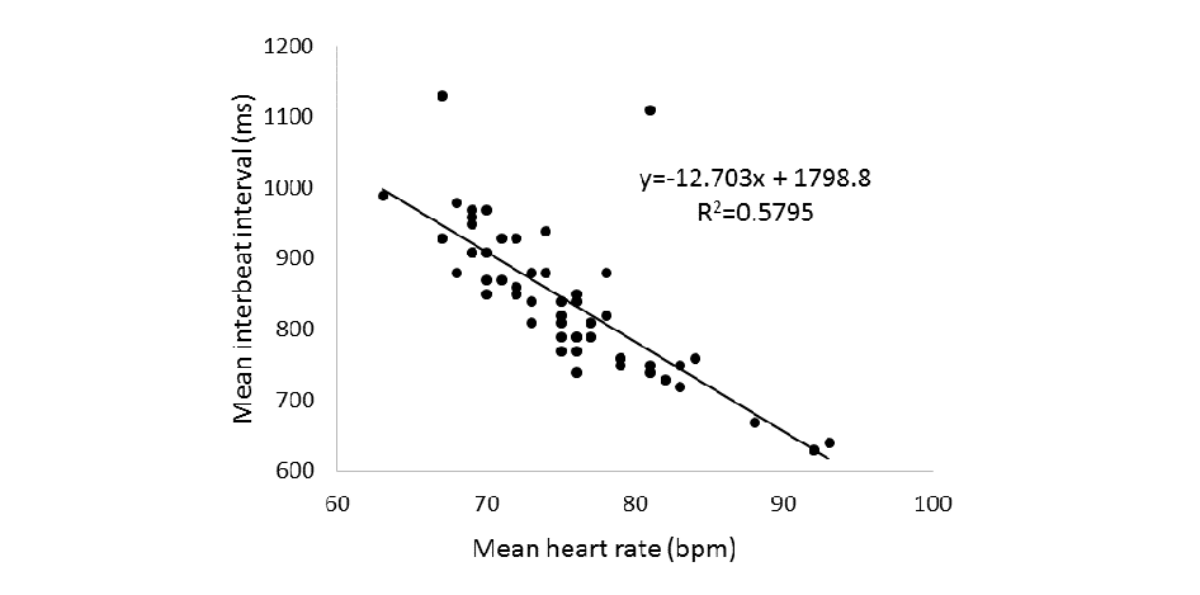
Scatterplot of the relationship between mean heart rate across the entire evaluation and mean interbeat interval length: n=52; Spearman correlation=−0.78; P≤.001. bpm: beats per minute.

We did not find significant differences for any variable in Table 1 between the individuals removed from correlational analyses and those that we retained (*P*=.17 to *P*=.99). Heart rate remained within a normal range during the baseline assessment: mean of 76 beats per minute (range 63-93). Mean heart rate over the course of the assessment was inversely correlated with the mean interbeat interval length ρ=−0.78 and *P*≤.001 (Figure 1) as expected, because a higher heart rate corresponds to a shorter duration between beats and vice versa.

**Figure 2:**
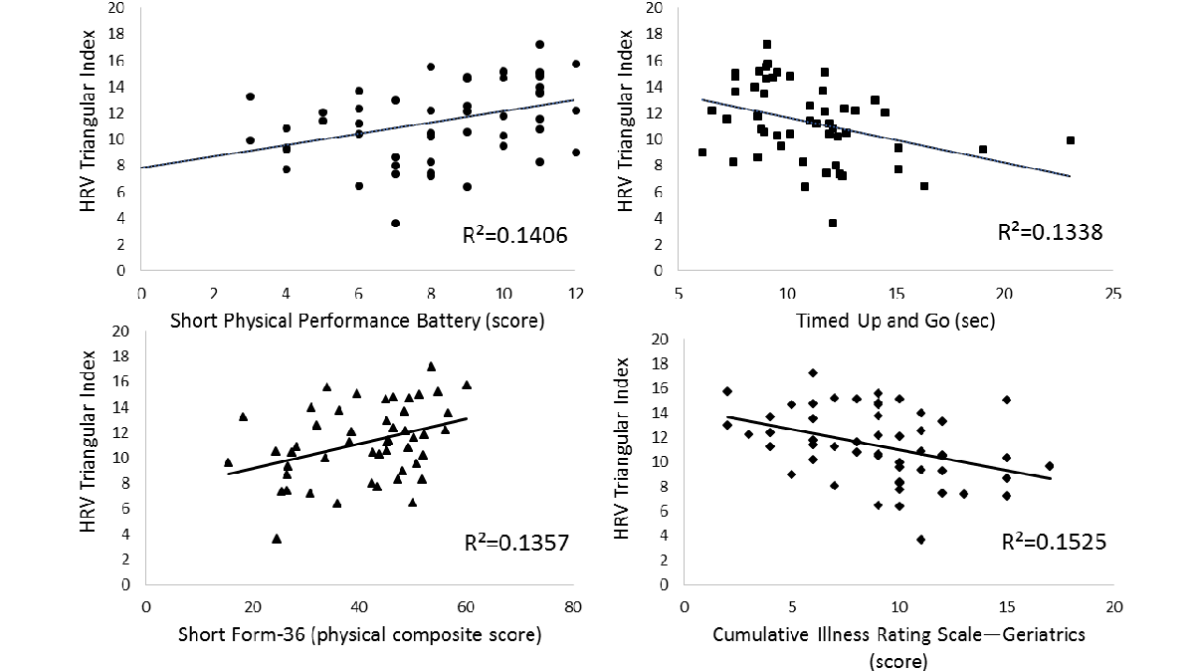
Scatter plots of the relationships between heart rate variability triangular index (TI) and (a) Short Physical Performance Battery scores (n=52), (b) Timed Up and Go scores (n=51), (c) Short Form 36 physical composite scores (n=49), and (d) Cumulative Illness Rating Scale for Geriatrics (CIRS-G) scores (n=52). HRV: heart rate variability; SF-36: Short Form 36.

There were no significant differences in the HRV TI based on sex (*U*=242.5; *P*=.40), antihypertensive medication use (*U*=271.5; *P*=.37), or antidepressant medication use (*U*=147.0; *P*=.59). We also did not observe significant correlations between any potential covariate and both HRV TI and physical function measures (ie, variables that could potentially modify these bivariate relationships; Table 2). Therefore, we did not adjust for any variables using partial correlations for our primary hypothesis regarding relationships between HRV TI and physical function measures. HRV TI was significantly related to SPPB, TUG, and SF-36 physical composite scores (Table 2 and Figure 2). We also observed an inverse relationship between HRV TI and CIRS-G scores (Figure 2).

Results of the regression revealed that the HRV TI explained a significant proportion of variance in physical health status as characterized by both SF-36 physical composite and CIRS-G scores. *R^2^* values increased from 0.11 to 0.28, with the HRV TI included in estimating SF-36 physical composite scores, and from 0.17 to 0.33, with the HRV TI included in estimating CIRS-G scores. Additional significant correlates for these measures of physical health status following the backward elimination procedure included gender, BMI, medication use, and smoking status (Table 3). VIF values were all less than 2.0, suggesting minimal collinearity.

**Table 1:** Sociodemographic information and other variables collected from participants.

Variable name		Values
Age (years; n=77), mean (SD); range		82.9 (6.7); 67-98
Gender (n=77); number of females, n (%)		52 (68)
Education (years; n=77), mean (SD); range		15.8 (2.4); 12-20
Race (n=77); number of whites, n (%)		68 (88)
Body mass index (kg/m^2^; n=76), mean (SD); range		27.9 (4.9); 19-43
Waist-to-hip ratio (ratio; n=75), mean (SD); range		0.87 (0.08); 0.71-1.06
Systolic blood pressure (mm Hg; n=74), mean (SD); range		134 (17); 100-167
Diastolic blood pressure (mm Hg; n=74), mean (SD); range		75 (9); 56-94
University of California San Diego Performance-Based Skills Assessment Brief (score; n=77), mean (SD); range		76.1 (12.5); 37.4-100.0
Cumulative Illness Rating Scale for Geriatrics (score; n=76), mean (SD); range		8.8 (3.2); 2-15
Antihypertensives (n=75); number of participants using, n (%)		48 (62)
Antidepressants (n=75); number of participants using, n (%)		13 (17)
Current smoker (n=75); number of participants smoking, n (%)		1 (1)
**Alcohol use (n=68), n (%)**		
	Lifetime abstainer (number of yes; [% abstaining])	6 (9)
	Current infrequent drinker (number of yes; [% drinking])	36 (53)
	Current regular drinker (number of yes; [% drinking])	17 (25)
	Former drinker (number of yes; [% used to drink])	9 (13)
Short Physical Performance Battery (score out of 12; n=76), mean (SD); range		8.2 (2.7); 0-12
Timed up and Go time (seconds; n=74), mean (SD; range)		11.0 (3.1); 6.1-23.0
Short Form 36 physical composite (score out of 100; n=70), mean (SD); range		41.8 (10.7); 18.3-60.1
Heart rate variability triangular index (n=53), mean (SD); range		11.4 (2.9); 3.7-17.2

**Table 2:** Bivariate correlations among all variables. Correlations for primary hypotheses are in italics.

Variables	ρ (*P* value)									
	SPPB^a^ (n=52)	TUG^b^ (n=51)	SF-36 phys comp^c^ (n=49)	CIRS-G^d^ (n=52)	Age (n=53)	BMI^e^ (n=51)	Waist to hip ratio (n=52)	Sys BP^f^ (n=50)	Dia BP^g^ (n=50)	Mean HR^h^ (n=53)
HRV TI^i^ (n=53)	*0.41*^j^*;* (*.003*)	−*0.40*^j^*;* (*.004*)	*0.37*^j^*;* (*.009*)	−0.43^j^; (.001)	−0.10; (.47)	0.07; (.64)	−0.20; (.16)	0.07; (.63)	0.01; (.97)	−0.36^j^; (.009)
SPPB	—^k^	−0.79^l^; (≤.001)	0.59^l^; (≤.001)	−0.23; (.11)	−0.29; (.04)	0.10; (.48)	−0.08; (.58)	0.06; (.69)	0.10; (.49)	−0.04; (.80)
TUG	—	—	−0.39^j^; (.007)	0.19; (.18)	0.40^j^; (.004)	−0.15; (.30)	0.10; (.50)	−0.02; (.87)	−0.13; (.39)	0.18; (.20)
SF-36 phys comp	—	—	—	−0.65^l^; (≤.001)	−0.24; (.10)	0.07; (.65)	−0.08; (.59)	−0.08; (.61)	−0.10; (.95)	−0.07; (.65)
CIRS-G	—	—	—	—	−0.20; (.16)	0.19; (.19)	−0.05; (.75)	−0.02; (.88)	−0.03; (.84)	0.09; (.54)
Age	—	—	—	—	—	−0.14; (.34)	0.40^l^; (.003)	0.35^j^; (.01)	0.18; (.21)	0.00; (>.99)
BMI	—	—	—	—	—	—	0.01; (.95)	−0.12; (.42)	0.13; (.37)	−0.21; (.14)
Waist to hip ratio	—	—	—	—	—	—	—	0.13; (.36)	0.05; (.74)	−0.09; (.51)
Sys BP	—	—	—	—	—	—	—	—	0.64^l^; (≤.001)	−0.11; (.46)
Dia BP	—	—	—	—	—	—	—	—	—	−0.03; (.85)

^a^SPPB: Short Physical Performance Battery.

^b^TUG: Timed Up and Go.

^c^SF-36 phys comp: Short Form 36 physical composite score.

^d^CIRS-G: Cumulative Illness Rating Scale for Geriatrics.

^e^BMI: body mass index.

^f^Sys BP: systolic blood pressure.

^g^Dia BP: diastolic blood pressure.

^h^HR: heart rate.

^i^HRV TI: heart rate variability triangular index.

^j^*P*≤.017.

^k^Not applicable.

^l^*P*≤.001.

**Table 3:** Results of the regression analyses (backward selection): remaining significant correlates of physical health status characterized by Short Form 36 physical composite scores and Cumulative Illness Rating Scale for Geriatrics scores. Adjusted R^2^ of the full model for SF-36 scores=0.28 and for CIRS-G scores=0.33.

Parameter		Estimate	Naïve SE	Naïve z	Robust SE	Robust z	*P* value
**SF-36^a^ physical composite score**							
	Intercept	22.01	5.81	3.79	6.05	3.64	≤.001
	HRV TI^b^	1.58	0.47	3.38	0.44	3.58	≤.001
	Gender	5.88	3.01	1.96	2.79	2.11	.04
	Antihypertensives	−5.95	2.71	−2.20	2.53	−2.35	.02
	Smoking status	6.72	2.71	2.48	2.46	2.73	.006
**CIRS-G^c^ score**							
	Intercept	8.62	3.02	2.85	2.80	3.07	.002
	HRV TI^d^	−0.46	0.14	−3.41	0.10	−4.56	≤.001
	Gender	−1.56	0.89	−1.75	0.81	−1.93	.05
	BMI^e^	0.15	0.09	1.70	0.07	1.96	.05
	Antihypertensives	2.52	0.83	3.05	0.71	3.57	≤.001
	Antidepressants	1.34	0.91	1.47	0.95	1.41	.16

^a^SF-36: short form 36.

^b^HRV TI: heart rate variability triangular index

^c^CIRS-G: Cumulative Illness Rating Scale for Geriatrics.

^d^HRV: heart rate variability.

^e^BMI: body mass index.

## Discussion

### Principal Findings

In this study, we explored relationships between HRV measured using a wrist-worn device and both capacity-based and self-reported measures of physical function obtained during a standard clinical geriatric assessment of older adults living independently in a CCSHC. Our primary hypothesis was supported as we observed significant relationships between the HRV TI and both capacity-based measures of physical function—that is, the SPPB and TUG [32-34] and self-reported physical function, as measured by the SF-36 physical composite score [35,43]. In addition, we observed that the HRV TI explained a significant proportion of variance in physical health status, as characterized by SF-36 physical composite and CIRS-G scores [40].

### Relationships Between Heart Rate Variability, Physical Function, and Physical Health

The relationships between the HRV TI and both the SPPB and TUG and self-reported measures of physical function are noteworthy. Self-reported and capacity-based measures of physical capacity are reported to contribute information about physical function in different ways [58]. For example, self-reported measures are more effective in distinguishing among persons at lower levels of physical function who may not be able to complete capacity-based tests [59]. In contrast, self-reported measures fail to differentiate well among persons in the mid- or high range of functioning. Capacity-based measures provide information across a broader range of physical function and discriminate more effectively than self-reported measures at higher capacities [59]. We observed significant relationships between SPPB and TUG scores and self-reported physical function in our sample. We also observed an inverse relationship between the presence of physical comorbidities and self-reported physical function, which is consistent with previous reports demonstrating that older adults who report more health problems also report lower levels of perceived function [60]. Presence of physical comorbidities (CIRS-G) was related to the HRV TI but not to either SPPB or TUG scores in this study. Therefore, HRV TI may characterize a complementary aspect of older adults’ physical health that is not captured by physical capacity measures.

Previous work has identified relationships between HRV quantified via standard ECG or Holter monitoring and aspects of older adults’ physical function [5,16,19]. In particular, frailty appears to be linked to a loss of physiologic flexibility, as quantified by nonlinear HRV dynamics [16,17]. If changes in HRV precede physical decline, then regular measurement of HRV may be a useful biomarker for older adults. Low HRV, along with male sex, older age, and smoking, has also been reported to be a significant predictor of chronic diseases including hypertension and hyperglycemia and a diagnosis of diabetes within 12 years [61]. These findings suggest that monitoring of HRV may help to identify individuals at high risk for both physical decline and development of chronic diseases.

High HRV has been described as a measure of adaptability [62], which is directly related to maintenance of physical function. The consistent relationships observed between the HRV TI and measures of physical function and physical health in this study suggest that HRV TI quantified with a wearable device is indeed related to physical function and cumulative illness burden in older adults. These relationships were not accounted for by a direct association between HRV and age, suggesting that this measure of autonomic function may be useful for aging research. This finding is not surprising given that previous relationships between HRV and age have generally been observed across a much broader age span [4,14,15,28]. Although standard ECG and Holter monitor recordings have been useful for risk stratification in a variety of pathologic conditions [62-64], our findings suggest that a wearable device may also be useful for quantifying autonomic dysfunction in older adults. Although HRV was significantly associated with physical capacity measures, mean heart rate was not, suggesting that measurement of heart rate alone does not capture the relationship between autonomic regulation and physical function.

### Wearable Technology for Heart Rate Variability Measurement

The accessibility of wearable technology [29] and the relationships between HRV derived from a wrist-worn wearable and measures of physical function suggest that incorporating wearables for HRV monitoring into clinical geriatric assessments could potentially help in the early detection of physical decline. In particular, watch-like devices are ideal because they are noninvasive and small and do not interfere with the activities performed during clinical geriatric health assessments. These devices are also relatively low cost, do not require expert setup, and may be more easily deployed than ECG equipment in remote home-health settings. In the future, such wearables may be ideal for monitoring the health of older adults, further enabling early detection of physical decline and early intervention to improve autonomic regulation and possibly delay deterioration of physical function. However, such long-term monitoring of HRV via wearable devices will necessitate careful consideration of the adoption of technology by older adults and ethical concerns such as data privacy and informed consent [65]. It may also be important to monitor HRV in younger populations, given the observed relationships to measures of physical health status in this study and the fact that declining physical function does not happen only in adults older than 65 years. HRV is known to decline with age, particularly before the age of 60 years, but these correlations are only modest [14]. Thus, perhaps those with poorer physical health also exhibit greater tendency for decline. If HRV metrics are related to the physical health status of younger adults as well, early identification of declining HRV may enable more timely intervention to mitigate further decline.

### Future Goals for Heart Rate Variability Measurement

In this study, we measured HRV during a single assessment to demonstrate relationships between HRV metrics quantified with a wearable device and clinical measures of physical function; however, we intend to track this cohort of participants longitudinally. These findings will help determine whether HRV from a wearable device predicts or coincides with changes in physical function. We are also interested in the feasibility of more frequent HRV measurements using wrist-worn devices for older adults and plan to examine day-to-day variability of HRV metrics. The clinical value of HRV measurement is likely to be at the level of the individual and not the group; thus, identifying changes outside of an individual’s normal HRV range may lend the most insight into incipient decline.

### Study Limitations

This study had several limitations. This was a small sample of predominantly white and middle-to-upper class older adults, so our findings may not apply to other populations. We also had a majority of female participants, which may have prevented us from detecting differences in HRV due to gender. However, gender differences in HRV have been reported to disappear after the age of 50 years [14]. Furthermore, the cross-sectional nature of this investigation precludes the ability to draw causal relationships between the variables investigated. We investigated only 1 PPG device, the Empatica E4 sensor, in this study. However, we did not use an HRV metric provided by this device (Empatica does not offer these data). We simply used the device to collect raw interbeat interval data and then used the well-known Kubios HRV software [48] to calculate HRV TI. The presence of missing interbeat intervals influenced our choice of HRV metric (we selected the HRV TI instead of others) and, in particular, prevented us from using frequency-based HRV metrics as they are affected by missing interbeat intervals. As frequency-based metrics are reported to be more descriptive of the balance between sympathetic and parasympathetic activity [66], improving the quality of interbeat interval recordings from wearable devices is of considerable importance. We also had to remove a considerable number of participants from statistical analyses because of poor-quality interbeat interval recordings. Future research directed at refining wearable technology such as the Empatica E4 to improve the quality of recordings (eg, further minimize movement artifacts) will be essential in improving the utility of these devices. Finally, we cannot easily compare the HRV TI values obtained in this study to previously established norms for several reasons, including less frequent reporting of the HRV TI metric, differences in calculation methods, and differing durations of recordings (eg, often done over a 24-hour period).

### Conclusions

In summary, we demonstrated that the HRV TI measured using a relatively novel wearable device, the Empatica E4 sensor, was related to both objective (SPPB and TUG) and self-reported (SF-36 physical composite) measures of physical function of older adults and cumulative illness burden (CIRS-G) collected during a single assessment. In addition, the HRV TI explained a significant proportion of variance in physical health status, as characterized by the SF-36 physical composite scores and CIRS-G scores, beyond typically measured aspects of physical health. The next steps include longitudinal tracking of changes in both HRV and physical function, which will add important insights regarding the possible predictive value of HRV as a biomarker of functional outcomes in older adults.

## Abbreviations

BMI: body mass index
CCHSC: continuing care senior housing community
CIRS-G: Cumulative Illness Rating Scale for Geriatrics
ECG: electrocardiogram
GEE: generalized estimating equation
HRPP: Human Research Protections Program
HRV: heart rate variability
PPG: photoplethysmography
SF-36: Short Form 36
SPPB: Short Physical Performance Battery
TI: triangular index
TUG: Timed Up and Go
VIF: variance inflation factor

## References

[1] Berntson GG, Bigger JT, Eckberg DL, Grossman P, Kaufmann PG, Malik M, Nagaraja HN, Porges SW, Saul JP, Stone PH, van der Molen MW (1997). Heart rate variability: origins, methods, and interpretive caveats. Psychophysiology.

[2] Ernst G (2017). Heart-rate variability-more than heart beats?. Front Public Health.

[3] Shaffer F, Ginsberg JP (2017). An overview of heart rate variability metrics and norms. Front Public Health.

[4] Almeida-Santos MA, Barreto-Filho JA, Oliveira JL, Reis FP, da Cunha OC, Sousa AC (2016). Aging, heart rate variability and patterns of autonomic regulation of the heart. Arch Gerontol Geriatr.

[5] McCraty R, Shaffer F (2015). Heart rate variability: new perspectives on physiological mechanisms, assessment of self-regulatory capacity, and health risk. Glob Adv Health Med.

[6] Thayer JF, Yamamoto SS, Brosschot JF (2010). The relationship of autonomic imbalance, heart rate variability and cardiovascular disease risk factors. Int J Cardiol.

[7] Fei LU, Keeling PJ, Sadoul N, Copie X, Malik M, McKenna WJ, Camm AJ (1996). Decreased heart rate variability in patients with congestive heart failure and chronotropic incompetence. Pacing Clin Electrophysiol.

[8] Kleiger RE, Stein PK, Bigger JT (2005). Heart rate variability: measurement and clinical utility. Ann Noninvasive Electrocardiol.

[9] Kemp AH, Quintana DS (2013). The relationship between mental and physical health: insights from the study of heart rate variability. Int J Psychophysiol.

[10] Williams DP, Cash C, Rankin C, Bernardi A, Koenig J, Thayer JF (2015). Resting heart rate variability predicts self-reported difficulties in emotion regulation: a focus on different facets of emotion regulation. Front Psychol.

[11] Jandackova VK, Scholes S, Britton A, Steptoe A (2016). Are changes in heart rate variability in middle-aged and older people normative or caused by pathological conditions? Findings from a large population-based longitudinal cohort study. J Am Heart Assoc.

[12] Reardon M, Malik M (1996). Changes in heart rate variability with age. Pacing Clin Electrophysiol.

[13] Santillo E, Migale M, Fallavollita L, Marini L, Balestrini F (2012). Electrocardiographic analysis of heart rate variability in aging heart. Advances in Electrocardiograms - Methods and Analysis.

[14] Umetani K, Singer DH, McCraty R, Atkinson M (1998). Twenty-four hour time domain heart rate variability and heart rate: relations to age and gender over nine decades. J Am Coll Cardiol.

[15] O'Brien IA, O'Hare P, Corrall RJ (1986). Heart rate variability in healthy subjects: effect of age and the derivation of normal ranges for tests of autonomic function. Br Heart J.

[16] Chaves PH, Varadhan R, Lipsitz LA, Stein PK, Windham BG, Tian J, Fleisher LA, Guralnik JM, Fried LP (2008). Physiological complexity underlying heart rate dynamics and frailty status in community-dwelling older women. J Am Geriatr Soc.

[17] Katayama PL, Dias DP, Silva LE, Virtuoso-Junior JS, Marocolo M (2015). Cardiac autonomic modulation in non-frail, pre-frail and frail elderly women: a pilot study. Aging Clin Exp Res.

[18] Koopman JJ, van Bodegom D, Maan AC, Li Z, Ziem JB, Westendorp RG, Jukema JW (2015). Heart rate variability, but not heart rate, is associated with handgrip strength and mortality in older Africans at very low cardiovascular risk: a population-based study. Int J Cardiol.

[19] Varadhan R, Chaves PH, Lipsitz LA, Stein PK, Tian J, Windham BG, Berger RD, Fried LP (2009). Frailty and impaired cardiac autonomic control: new insights from principal components aggregation of traditional heart rate variability indices. J Gerontol A Biol Sci Med Sci.

[20] (1996). Heart rate variability. Standards of measurement, physiological interpretation, and clinical use. Task Force of the European Society of Cardiology and the North American Society of Pacing and Electrophysiology. Eur Heart J.

[21] Parak J, Korhonen I (2014). Evaluation of wearable consumer heart rate monitors based on photopletysmography. Conf Proc IEEE Eng Med Biol Soc.

[22] Peake JM, Kerr G, Sullivan JP (2018). A critical review of consumer wearables, mobile applications, and equipment for providing biofeedback, monitoring stress, and sleep in physically active populations. Front Physiol.

[23] Kamišalić A, Fister I, Turkanović M, Karakatič S (2018). Sensors and functionalities of non-invasive wrist-wearable devices: a review. Sensors (Basel).

[24] Tamura T, Maeda Y, Sekine M, Yoshida M (2014). Wearable photoplethysmographic sensors—past and present. Electronics.

[25] Georgiou K, Larentzakis AV, Khamis NN, Alsuhaibani GI, Alaska YA, Giallafos EJ (2018). Can wearable devices accurately measure heart rate variability? A systematic review. Folia Med (Plovdiv).

[26] Gil E, Orini M, Bailón R, Vergara JM, Mainardi L, Laguna P (2010). Photoplethysmography pulse rate variability as a surrogate measurement of heart rate variability during non-stationary conditions. Physiol Meas.

[27] Schäfer A, Vagedes J (2013). How accurate is pulse rate variability as an estimate of heart rate variability? A review on studies comparing photoplethysmographic technology with an electrocardiogram. Int J Cardiol.

[28] Ziegler D, Piolot R, Strassburger K, Lambeck H, Dannehl K (1999). Normal ranges and reproducibility of statistical, geometric, frequency domain, and non-linear measures of 24-hour heart rate variability. Horm Metab Res.

[29] Castaneda D, Esparza A, Ghamari M, Soltanpur C, Nazeran H (2018). A review on wearable photoplethysmography sensors and their potential future applications in health care. Int J Biosens Bioelectron.

[30] Garbarino M, Lai M, Bender D, Picard RW, Tognetti S (2014). Empatica E3 — A Wearable Wireless Multi-Sensor Device for Real-Time Computerized Biofeedback and Data Acquisition. Proceedings of the 4th International Conference on Wireless Mobile Communication and Healthcare - Transforming Healthcare Through Innovations in Mobile and Wireless Technologies.

[31] Mccarthy C, Pradhan N, Redpath C, Adler A (2016). Validation of the Empatica E4 Wristband. Proceedings of the EMBS International Student Conference.

[32] Guralnik JM, Simonsick EM, Ferrucci L, Glynn RJ, Berkman LF, Blazer DG, Scherr PA, Wallace RB (1994). A short physical performance battery assessing lower extremity function: association with self-reported disability and prediction of mortality and nursing home admission. J Gerontol.

[33] Guralnik JM, Ferrucci L, Pieper CF, Leveille SG, Markides KS, Ostir GV, Studenski S, Berkman LF, Wallace RB (2000). Lower extremity function and subsequent disability: consistency across studies, predictive models, and value of gait speed alone compared with the short physical performance battery. J Gerontol A Biol Sci Med Sci.

[34] Podsiadlo D, Richardson S (1991). The timed 'Up & Go': a test of basic functional mobility for frail elderly persons. J Am Geriatr Soc.

[35] McHorney CA, Ware JE, Raczek AE (1993). The MOS 36-item short-form health survey (SF-36): II. Psychometric and clinical tests of validity in measuring physical and mental health constructs. Med Care.

[36] Ward KT, Reuben DB (2003). UpToDate.

[37] Roedl KJ, Wilson LS, Fine J (2016). A systematic review and comparison of functional assessments of community-dwelling elderly patients. J Am Assoc Nurse Pract.

[38] Studenski S, Perera S, Wallace D, Chandler JM, Duncan PW, Rooney E, Fox M, Guralnik JM (2003). Physical performance measures in the clinical setting. J Am Geriatr Soc.

[39] Freiberger E, de Vreede P, Schoene D, Rydwik E, Mueller V, Frändin K, Hopman-Rock M (2012). Performance-based physical function in older community-dwelling persons: a systematic review of instruments. Age Ageing.

[40] Parmelee PA, Thuras PD, Katz IR, Lawton MP (1995). Validation of the cumulative illness rating scale in a geriatric residential population. J Am Geriatr Soc.

[41] Jeste DV, Glorioso D, Lee EE, Daly R, Graham S, Liu J, Paredes AM, Nebeker C, Tu XM, Twamley EW, van Patten R, Yamada Y, Depp C, Kim H (2019). Study of independent living residents of a continuing care senior housing community: sociodemographic and clinical associations of cognitive, physical, and mental health. Am J Geriatr Psychiatry.

[42] Mausbach BT, Harvey PD, Goldman SR, Jeste DV, Patterson TL (2007). Development of a brief scale of everyday functioning in persons with serious mental illness. Schizophr Bull.

[43] Hays RD, Sherbourne CD, Mazel RM (1993). The RAND 36-item health survey 1.0. Health Econ.

[44] Baek HJ, Shin JW (2017). Effect of missing inter-beat interval data on heart rate variability analysis using wrist-worn wearables. J Med Syst.

[45] Kos M, Li X, Khaghani-Far I, Gordon CM, Pavel M, Jimison HB (2017). Can accelerometry data improve estimates of heart rate variability from wrist pulse PPG sensors?. Conf Proc IEEE Eng Med Biol Soc.

[46] Zhang Z, Pi Z, Liu B (2015). TROIKA: a general framework for heart rate monitoring using wrist-type photoplethysmographic signals during intensive physical exercise. IEEE Trans Biomed Eng.

[47] Renevey P, Vetter R, Krauss J, Celka P, Depeursinge Y (2001). Wrist-located Pulse Detection Using IR Signals, Activity and Nonlinear Artifact Cancellation. Conference Proceedings of the 23rd Annual International Conference of the IEEE Engineering in Medicine and Biology Society.

[48] Tarvainen MP, Niskanen JP, Lipponen JA, Ranta-Aho PO, Karjalainen PA (2014). Kubios HRV--heart rate variability analysis software. Comput Methods Programs Biomed.

[49] Tarvainen MP, Niskanen JP (2012). Heart Rrate Variability - Kubios HRV.

[50] Tognetti S, Cenci I, Resnati D, Garbarino M, Lai M (2017). United States Patent.

[51] Kim KK, Lim YG, Kim JS, Park KS (2007). Effect of missing RR-interval data on heart rate variability analysis in the time domain. Physiol Meas.

[52] Kim KK, Kim JS, Lim YG, Park KS (2009). The effect of missing RR-interval data on heart rate variability analysis in the frequency domain. Physiol Meas.

[53] Jovic A, Bogunovic N (2011). Electrocardiogram analysis using a combination of statistical, geometric, and nonlinear heart rate variability features. Artif Intell Med.

[54] Antelmi I, de Paula RS, Shinzato AR, Peres CA, Mansur AJ, Grupi CJ (2004). Influence of age, gender, body mass index, and functional capacity on heart rate variability in a cohort of subjects without heart disease. Am J Cardiol.

[55] Searle SR (1997). Linear Models. First Edition.

[56] Tang W, He HT, Tu XM (2012). Applied Categorical and Count Data Analysis.

[57] Wang H, Peng J, Wang B, Lu X, Zheng JZ, Wang K, Tu XM, Feng C (2017). Inconsistency between univariate and multiple logistic regressions. Shanghai Arch Psychiatry.

[58] Prince SA, Adamo KB, Hamel ME, Hardt J, Gorber SC, Tremblay M (2008). A comparison of direct versus self-report measures for assessing physical activity in adults: a systematic review. Int J Behav Nutr Phys Act.

[59] Kasper JD, Chan KS, Freedman VA (2017). Measuring physical capacity: an assessment of a composite measure using self-report and items. J Aging Health.

[60] Daltroy LH, Larson MG, Eaton HM, Phillips CB, Liang MH (1999). Discrepancies between self-reported and observed physical function in the elderly: the influence of response shift and other factors. Soc Sci Med.

[61] Wulsin LR, Horn PS, Perry JL, Massaro JM, D'Agostino RB (2015). Autonomic imbalance as a predictor of metabolic risks, cardiovascular disease, diabetes, and mortality. J Clin Endocrinol Metab.

[62] Thayer JF, Ahs F, Fredrikson M, Sollers JJ, Wager TD (2012). A meta-analysis of heart rate variability and neuroimaging studies: implications for heart rate variability as a marker of stress and health. Neurosci Biobehav Rev.

[63] Chandra P, Sands RL, Gillespie BW, Levin NW, Kotanko P, Kiser M, Finkelstein F, Hinderliter A, Pop-Busui R, Rajagopalan S, Saran R (2012). Predictors of heart rate variability and its prognostic significance in chronic kidney disease. Nephrol Dial Transplant.

[64] Farrell TG, Bashir Y, Cripps T, Malik M, Poloniecki J, Bennett ED, Ward DE, Camm AJ (1991). Risk stratification for arrhythmic events in postinfarction patients based on heart rate variability, ambulatory electrocardiographic variables and the signal-averaged electrocardiogram. J Am Coll Cardiol.

[65] Kang HG, Mahoney DF, Hoenig H, Hirth VA, Bonato P, Hajjar I, Lipsitz LA, Center for Integration of Medicine and Innovative Technology Working Group on Advanced Approaches to Physiologic Monitoring for the Aged (2010). In situ monitoring of health in older adults: technologies and issues. J Am Geriatr Soc.

[66] Singh N, Moneghetti KJ, Christle JW, Hadley D, Plews D, Froelicher V (2018). Heart rate variability: an old metric with new meaning in the era of using mhealth technologies for health and exercise training guidance. Part one: physiology and methods. Arrhythm Electrophysiol Rev.

